# Advances in Biomaterials for the Prevention and Disruption of *Candida* Biofilms

**DOI:** 10.3389/fmicb.2020.538602

**Published:** 2020-09-17

**Authors:** Noel Vera-González, Anita Shukla

**Affiliations:** ^1^Center for Biomedical Engineering, School of Engineering, Brown University, Providence, RI, United States; ^2^Institute for Molecular and Nanoscale Innovation, Brown University, Providence, RI, United States

**Keywords:** *Candida*, biofilms, biomaterials, antifungal, surface functionalization, nanoparticles, antifungal polymers

## Abstract

*Candida* species can readily colonize a multitude of indwelling devices, leading to biofilm formation. These three-dimensional, surface-associated *Candida* communities employ a multitude of sophisticated mechanisms to evade treatment, leading to persistent and recurrent infections with high mortality rates. Further complicating matters, the current arsenal of antifungal therapeutics that are effective against biofilms is extremely limited. Antifungal biomaterials are gaining interest as an effective strategy for combating *Candida* biofilm infections. In this review, we explore biomaterials developed to prevent *Candida* biofilm formation and those that treat existing biofilms. Surface functionalization of devices employing clinically utilized antifungals, other antifungal molecules, and antifungal polymers has been extremely effective at preventing fungi attachment, which is the first step of biofilm formation. Several mechanisms can lead to this attachment inhibition, including contact killing and release-based killing of surrounding planktonic cells. Eliminating mature biofilms is arguably much more difficult than prevention. Nanoparticles have shown the most promise in disrupting existing biofilms, with the potential to penetrate the dense fungal biofilm matrix and locally target fungal cells. We will describe recent advances in both surface functionalization and nanoparticle therapeutics for the treatment of *Candida* biofilms.

## Introduction

*Candida* is one of the most common causes of fungal infections worldwide, responsible for over 400,000 infections per year (Brown et al., 2012; Tsui et al., 2016). A commensal fungus that can readily become pathogenic, *Candida*, is known to form biofilms (Gulati and Nobile, 2016). These surface-attached, three-dimensional communities of tightly packed fungi can serve as infection strongholds, complicating treatment and leading to persistent fungemia (Li et al., 2018). *Candida* biofilm related infections have mortality rates as high as 41% (Rajendran et al., 2016). Biofilms protect fungal cells from the host immune system and often increase drug resistance (Mukherjee and Chandra, 2004; Nett, 2014). Biofilm fungi secrete a dense extracellular polymeric substance (EPS) that acts as a physical barrier for antifungal therapeutics, most of which are hydrophobic with limited ability to penetrate this matrix (Singh et al., 2018). Persister cells, which are metabolically dormant, can form as quickly as cell attachment occurs, leading to changes in gene expression, with an initial overexpression of drug efflux pumps, followed by a reduction in membrane sterol content in mature *Candida* biofilms (Kumamoto and Vinces, 2005; LaFleur et al., 2006). Although persister cells represent a small subpopulation within the biofilm (~1% of all cells), their tolerance to high doses of antimicrobials allows them to readily repopulate the biofilm once the treatment has stopped, resulting in recurring infections (Galdiero et al., 2020). Quorum sensing can mediate the secretion of signaling factors affecting *Candida* gene expression and behavior, including filamentation (Mallick and Bennett, 2013; Tsui et al., 2016). Changes to the cell wall that enhance drug resistance can also occur; for example, cell walls that are twice as thick as planktonic cells have been observed in biofilm *Candida* (Nett et al., 2007; Lima et al., 2019).

**Figure 1:**
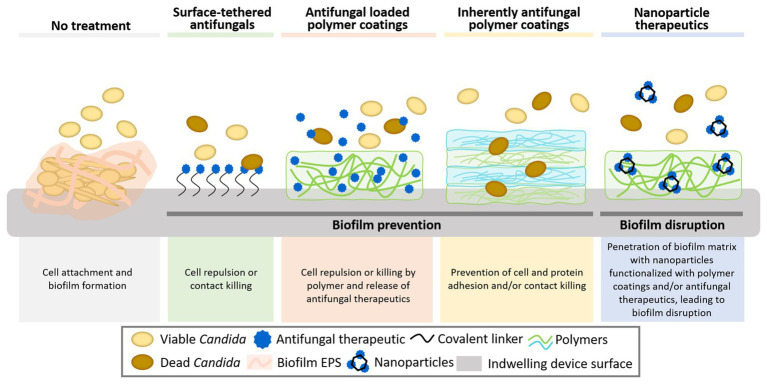
Biomaterials strategies to combat surface-associated *Candida* biofilms. These strategies include direct surface functionalization with antimicrobial small molecules and natural and synthetic polymers and the use of nanoparticles, which may better penetrate the dense biofilm matrix and potentially target fungal cells. Together these strategies can prevent biofilm formation by inhibiting the initial attachment of fungi to surfaces and eradicate existing biofilms.

The majority of biofilm-associated *Candida* infections arise from cells that colonize the surfaces of implanted medical devices (Coad et al., 2016). These devices range from plastic cochlear implants and subcutaneous drug delivery devices, silicone or polyurethane catheters, and acrylic dental implants, to titanium hip implants, glass-ceramics used in bone repair, metal pacemakers, and polymeric contact lenses among many others (Vargas-Blanco et al., 2017; Cavalheiro and Teixeira, 2018; Devadas et al., 2019). Treatments for these biofilm-associated infections are extremely limited, with only three primary antifungal drug classes (polyenes, azoles, and echinocandins) and a total of 21 United States Food and Drug Administration (FDA) approved antifungal drugs (Butts and Krysan, 2012; McKeny and Zito, 2020), of which only a subset have demonstrated some level of antibiofilm activity. Innovations in biomaterials have the potential to combat *Candida* biofilms (Figure 1). Here, we explore recent promising approaches in this field involving surface modification with antifungal small molecules and polymers aimed at preventing biofilm formation and the design of nanoparticles aimed at both preventing and disrupting *Candida* biofilms.

## Preventing *Candida* Biofilms Using Surface Modification With Clinically Utilized Antifungals

**Figure 2:**
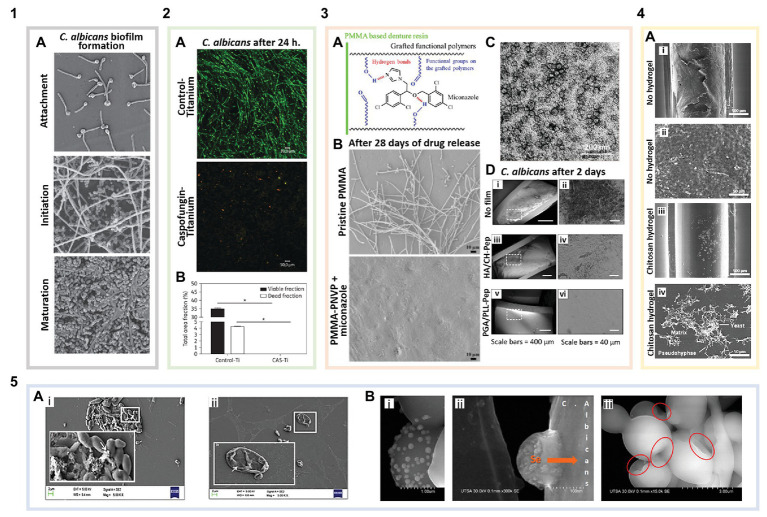
Biomaterials for the prevention and treatment of *Candida* biofilms. **(1)** Biofilm formation of non-functionalized surfaces: **(1A)** *Candida albicans* biofilm formation (adapted with permission from Ramage et al., 2009). **(2)** Surface-tethered antifungals: **(2A)** Live/Dead staining showing caspofungin functionalized titanium disks (with caspofungin surface coverage of ~2,191 pmol/cm^2^) inhibiting *C. albicans* attachment and biofilm formation compared to bare titanium (green are live cells, and red indicates membrane compromised cells; adapted with permission from Kucharíková et al., 2016). **(2B)** Quantification of viable cells per area in the images shown in 2A (adapted with permission from Kucharíková et al., 2016). **(3)** Antifungal loaded polymer coatings: **(3A)** Schematic illustrating miconazole-polymer hydrogen bonding (adapted with permission from Wen et al., 2016a). **(3B)** Miconazole loaded into poly(methyl methacrylate)-poly(1-vinyl-2-pyrrolidinone) (PMMA-PNVP) films inhibits *C. albicans* attachment and biofilm growth for up to 28 days compared to pristine PMMA (adapted with permission from Wen et al., 2016a). **(3C)** Antifungal poly(ethylene glycol) (PEG) + curcumin (CU) nanocomposites in solution after being released from graphene oxide (GO) coatings (adapted with permission from Devadas et al., 2019). **(3D)** Layer-by-layer (LbL) coated catheters prevent *C. albicans* attachment and biofilm formation after 2 days. **(i)** Uncoated catheters showing *C. albicans* attachment and biomass deposition. **(ii)** Magnified region outlined in **3D(i)**. **(iii)** Catheters coated with a hyaluronic acid (HA)/chitosan (CH) LbL film with β-peptide showing no *C. albicans* attachment but some biomass deposition on the surface. **(iv)** Magnified region outlined in **3D(iii)**. **(v)** Poly-L-glutamic acid (PGA)/poly-L-lysine (PLL) LbL film with β-peptide coated catheters showing no cell or biomass attachment. **(vi)** Magnified region outlined in **3D(v)** (adapted with permission from Raman et al., 2016). **(4)** Inherently antifungal polymer coatings: **(4A)** Polyurethane catheter-associated *Candida parapsilosis* biofilms. **(i)** Uncoated catheters exhibiting *Candida* attachment and biofilm formation. **(ii)** Magnified region of image **4A(i)** showing a dense *C. parapsilosis* biofilm. **(iii)** Catheters coated with low molecular weight CH hydrogels significantly reduce *Candida* cell attachment and biofilm formation. **(iv)** Magnified region of image **4A(iii)** showing biofilm disruption (adapted with permission from Silva-Dias et al., 2014). **(5)** Antifungal nanoparticles: **(5A)** Scanning electron microscopy (SEM) images of *C. albicans* biofilms on polystyrene. **(i)** Control biofilm cells [white arrow points to extracellular polymeric substances (EPSs)] and **(ii)** biofilm inhibition in the presence of ferulic acid-chitosan nanoparticles (white arrow indicates the damaged fungal cell wall; adapted with permission from Panwar et al., 2016). **(5B)** SEM images of selenium nanoparticles **(i,ii)** binding to and **(iii)** disrupting *C. albicans* cells in biofilms. The red circles indicate areas of the cell membrane, where the nanoparticles have induced shrinking and folding (adapted with permission from Guisbiers et al., 2017).

Inhibiting *Candida* attachment to surfaces, the first step of biofilm formation (Figure 2: 1A), is often the most effective way to combat biofilm-associated infections. Various approaches have been investigated to prevent fungi attachment, including surface functionalization with FDA-approved antifungals using covalent and non-covalent interactions (Zelikin, 2010). Caspofungin, the only echinocandin with primary amines, is most commonly used in direct surface tethering (Coad et al., 2015; Michl et al., 2017). Caspofungin tethered titanium disks cultured with *Candida albicans* showed complete inhibition of fungal attachment compared to bare titanium (Figure 2: 2A,B). These same caspofungin-tethered disks implanted subcutaneously into the backs of mice and challenged with *C. albicans* showed 89% less *Candida* attached after 2 days compared to bare disks (Kucharíková et al., 2016).

In an example of non-covalent drug tethering, β-cyclodextrins (CD) were grafted to polyethylene and polypropylene surfaces (Nava-Ortiz et al., 2010), commonly used in medical devices. CDs were used to promote host-guest interactions with the hydrophobic antifungal, miconazole, while also regulating interactions with proteins and increasing hemocompatibility. These miconazole loaded CD grafted surfaces exhibited up to a 97% reduction in the amount of recovered *C. albicans* compared to a silicone control incubated with the fungus. Polymers are also commonly used to enable non-covalent functionalization with antifungals, due to their ability to form multivalent interactions promoting loading of antifungal compounds. Wen et al. grafted poly(2-hydroxyethyl methacrylate) (PHEMA) onto poly(methyl methacrylate) (PMMA) denture resins. There is great interest in preventing *Candida* biofilms on dental surfaces, including dentures given the prevalence of *Candida* in the oral microbiota; in fact, *Candida* is responsible for up to 67% of denture-associated stomatitis (Ramage et al., 2006). PHEMA grafting was used to load the antifungal, clotrimazole, mediated *via* hydrogen bonding interactions, leading to a clotrimazole surface coverage of up to 46.0 ± 3.2 μg/cm^2^ compared to 5.2 ± 0.4 μg/cm^2^ on bare PMMA. A sustained release of clotrimazole was observed from the PHEMA-grafted denture disks over 28 days, yielding approximately a 50 and 36% reduction in *C. albicans* adhesion after 1 and 28 days, respectively, compared with non PHEMA-grafted disks (Wen et al., 2016b). Grafting poly(1-vinyl-2-pyrrolidinone) (PNVP) to PMMA enabled miconazole loading of 127.0 ± 15.1 μg/cm^2^, likely mediated *via* hydrophobic interactions and hydrogen bonding (Figure 2: 3A). PNVP-grafted resins with miconazole showed no *Candida* adhesion even after 28 days of drug release (Figure 2: 3B; Wen et al., 2016a). Along with superior biocompatibility, these functionalized materials can be used for extended biofilm prevention and have the potential to be reloaded with therapeutics.

## Preventing *Candida* Biofilms Using Surface Modification With New Antifungal Small Molecules and Peptides

Although promising, surface functionalization with FDA-approved antifungals raises concerns for increased resistance to these therapeutics. Thus, there is an interest in alternative approaches to prevent *Candida* biofilms utilizing non-clinically used small molecules and peptides with inherent antifungal and antibiofilm properties. One example, filastatin, a potent small molecule inhibitor of *Candida* attachment and filamentation was recently identified in a screen of 30,000 compounds (Fazly et al., 2013). Vargas-Blanco et al. (2017) found that incubation of *C. albicans* with various biomaterials in the presence of filastatin can inhibit *Candida* attachment to these materials. Adsorption of filastatin on dental resin and silicone showed that *Candida* cell attachment was reduced on these materials by 62.7 and 79.7%, respectively, compared to uncoated controls. By incorporating filastatin into the silicone matrix during polymerization a 6.5-fold reduction in *C. albicans* adhesion compared to untreated silicone controls was observed (Vargas-Blanco et al., 2017). Other small molecule biofilm inhibitors specifically interrupt *Candida* quorum sensing. These molecules include furanones, which are plant synthesized compounds that prevent microbial fouling on the plant surface. Devadas et al. (2019) coated common catheter materials with a furanone embedded polycaprolactone matrix. These polymer coatings retained 85% or more of the total loaded furanone over at least 30 days in solution. The attachment of clinical isolates of *Candida tropicalis*, *Candida glabrata*, and *Candida krusei* on these coated catheters was completely inhibited as determined by scanning electron microscopy (SEM; Devadas et al., 2019). Other plant derived compounds have also shown activity against *Candida* biofilms when combined with biomaterials. Recently, clove oil and red thyme oil incorporated in polycaprolactone electrospun nanofibers led to a 60 and 80% reduction in *C. tropicalis* attachment, respectively (Sahal et al., 2019). Initial results with these small molecules are promising; future studies will likely examine functionalization *via* covalent tethering or affinity-based interactions with these compounds to enable long-term antibiofilm activity.

Combination approaches to prevent *Candida* biofilms involving the inhibition of fungal cell attachment and simultaneous killing of planktonic fungi have also been investigated. Palmieri et al. (2018) developed a multilayered coating by drop-casting graphene oxide (GO) on polyurethane catheters, followed by curcumin (CU) and poly(ethylene glycol) (PEG). The GO was included to prevent *C. albicans* attachment due to its ability to generate oxidative stress and physically disrupt the cell wall and membrane. CU + PEG self-assembled nanocomposites (75–125 nm in diameter; Figure 2: 3C) were released from these coatings inhibiting planktonic *C. albicans* growth, with a minimum inhibitory concentration of 10.6 μg/ml. The complete catheter coating inhibited *C. albicans* attachment *in vitro* after 24 h with less than 20% biofilm formation compared to uncoated controls (Palmieri et al., 2018).

As an alternative to solvent casting or vapor deposition approaches, many biomedical surfaces have been coated *via* layer-by-layer (LbL) self-assembly to develop antifungal coatings. LbL assembly is a multilayer film fabrication method that involves alternating the adsorption of molecules and macromolecules (e.g., polyelectrolytes, peptides, proteins, small molecules, etc.,) with complementary functionalities most commonly by dip coating (Shukla and Almeida, 2014; Alkekhia and Shukla, 2019; Alkekhia et al., 2020). LbL films have been combined with antifungal peptides to exhibit remarkable antibiofilm properties. Antifungal peptides are considered potent and broad-spectrum antifungals; due to their multiple mechanisms of action, fungi are often unable to develop resistance to these peptides (Oshiro et al., 2019). These peptides are most commonly amphiphilic and cationic allowing them to readily interact with the fungal cell membrane, causing cell death (Karlsson et al., 2010). Raman et al. (2016) assembled an LbL film with hyaluronic acid (HA) and chitosan (CH) on catheter surfaces and used it as a reservoir for a synthetic antifungal β-peptide. The luminal surface of polyurethane catheters coated with these LbL films without any β-peptide was able to reduce viable *C. albicans* by approximately 25-fold after 6 h of exposure when compared to uncoated polyurethane catheters, demonstrating the innate antifungal properties of this polymeric coating. When passively loaded with the antifungal β-peptide, sustained release of the β-peptide was achieved over 50 days with complete eradication of planktonic *C. albicans in vitro*. Catheters coated with the β-peptide-loaded films tested in a rat central venous catheter model exhibited almost no fungal cells following 2 days [Figure 2: 3D(i–iv)]. However, this coated surface was found to contain a network of host proteins, which can yield complications, including fouling with red blood cells, which can stimulate platelet production. Another film architecture examined in the same study utilizing β-peptide-loaded poly-L-lysine (PLL) and poly-L-glutamic acid (PGA) LbL films exhibited both a complete lack of *Candida* cell attachment and host proteins when tested in the same *in vivo* model [Figure 2: 3D(v,vi)], emphasizing the importance of polymer choice in preventing overall fouling (Raman et al., 2016). PMMA denture disks were also recently coated with an LbL film containing the cationic mammalian salivary antifungal peptide, histatin-5 (H-5), and HA with a final H-5 layer. SEM images confirmed over 4 weeks that these LbL coated surfaces were able to completely prevent *Candida* attachment (Wen et al., 2018).

Many other small molecules and peptides not yet incorporated into biomaterials have demonstrated antibiofilm activity. Among these are newly synthesized imidazole derivatives, which have been found to prevent *Candida* biofilm formation and disrupt existing biofilms (Ribeiro et al., 2014; Gabriel et al., 2019). Thiazolylhydrazone derivatives have also recently emerged as effective antifungal compounds with low mammalian cell toxicity (Cruz et al., 2018). 2,6-Bis[(E)-(4-pyridyl)methylidene]cyclohexanone, an antiparasitic compound, was also found to exhibit antifungal properties including the inhibition of *Candida* filamentation, crucial in biofilm formation (de Sá et al., 2018). Antifungal peptide derivatives of H-5 are also being explored (Sultan et al., 2019), and other host defense peptides such as innate defense regulator 1018 and porcine cathelicidins have recently been shown to possess antifungal and antibiofilm properties against *Candida* (Lyu et al., 2016; Freitas et al., 2017). These compounds are potential candidates for incorporation into antifungal biomaterials.

## Preventing *Candida* Biofilms Using Polymer-Only Coatings

Many polymers themselves possess inherent antifouling, antifungal, and/or antibiofilm properties, while being less susceptible to resistance compared with small molecule antifungals; therefore, the use of polymer-only coatings for combating *Candida* biofilms has gained significant interest. For example, chitosan, a naturally derived polysaccharide, has been widely incorporated into hydrogels and coatings to prevent *Candida* attachment and biofilm formation (Carlson et al., 2008; Ailincai et al., 2016; Tan et al., 2016). It is hypothesized that chitosan interacts electrostatically *via* its positively charged amino groups with anionic moieties on microbial species leading to increased membrane permeability and eventual cell death (Jung et al., 2020). In a recent study, polyurethane intravenous catheters were coated with low molecular weight (50 kDa) chitosan hydrogels, implanted subcutaneously into mice, and subsequently challenged with *Candida parapsilosis*. Following 7 days, the chitosan-coated catheters reduced *Candida* metabolic activity by ~96% when compared to uncoated catheters, showcasing the ability of polymer-only coatings free of small molecule antifungals to achieve excellent antibiofilm activity. Reduced biomass on these chitosan coated catheters was shown using SEM (Figure 2: 4A; Silva-Dias et al., 2014). Chitosan has also been modified to enhance its antibiofilm properties. Jung et al. (2019) examined the use of amphiphilic quaternary ammonium chitosans (AQACs) in LbL coatings. LbL films containing sodium alginate and AQAC, effectively prevented cell attachment on coated PMMA substrates (Jung et al., 2019). AQACs have been shown to disrupt mature *Candida* biofilms by interacting electrostatically with the negatively charged biofilm surface (Jung et al., 2020). Coatings with other polymers including imidazolium salt (IS) conjugated poly(L-lactide) (PLA) have also been used to effectively prevent *Candida* attachment on coated surfaces (Schrekker et al., 2016).

## Nanoparticles for the Prevention of Fungal Cell Attachment and Biofilm Eradication

Despite the progress that has been made in antifungal surface functionalization, these approaches are limited in their ability to treat mature biofilms. Nanoparticles are a promising strategy to eradicate existing biofilms, with the potential to carry, stabilize, and protect therapeutic payloads, penetrate the EPS, target fungal cells, and be internalized (Ikuma et al., 2015; Qayyum and Khan, 2016; Stone et al., 2016). Several strategies have been used to develop nanoparticles for the treatment of fungal infections, from using inorganic compounds to antimicrobial polymers (Ahmad et al., 2016; Amaral et al., 2019). In an example of the latter approach, chitosan nanoparticles (20–30 nm diameter) were recently examined for their ability to inhibit *C. albicans* biofilm growth, following initial cell attachment. Incubation with chitosan nanoparticles for 3 h led to a greater than 50% reduction in biofilm mass compared to non-treated controls (Ikono et al., 2019). While these chitosan nanoparticles exhibited some inherent antibiofilm activity, they were unable to entirely inhibit or disrupt *Candida* biofilms. Panwar et al. (2016) instead incorporated ferulic acid, a plant derived small molecule with known antibiofilm properties (Teodoro et al., 2015), into chitosan nanoparticles (~115 nm diameter). On its own, ferulic acid cannot efficiently penetrate fungal biofilms; however, when incorporated into chitosan nanoparticles and incubated with *C. albicans* biofilms, a significant reduction in fungal metabolic activity was observed (22.5% normalized to an untreated biofilm following 24 h). While the mechanism of these nanoparticles is not fully understood, it is believed that their strong cationic surface charge allows them to localize to and disrupt the fungal cell membrane while the surface bound ferulic acid interrupts *Candida* oxidative phosphorylation. This cell damage is evident in SEM images [Figure 2: 5A(ii)] when compared to healthy biofilm cells [Figure 2: 5A(i); Panwar et al., 2016].

Lipid-based self-assembled nanoparticles have also shown promise in penetrating the biofilm matrix and in targeting fungal cells. AmBisome®, a widely utilized liposomal formulation of amphotericin B, is able to disrupt *Candida* biofilms while free amphotericin B is unable to do this (Stone et al., 2016). AmBisome has a strong affinity for *Candida* cells, electrostatically interacting with the cell wall before binding to the cell membrane at sites of high ergosterol content (Soo Hoo, 2017), which may promote their activity against biofilm *Candida* cells, which have been shown to have thicker cell walls (Nett et al., 2007). Liposomal amphotericin B has also been immobilized on biomaterial surfaces for the prevention of biofilm formation (Alves et al., 2019). In our recent work, we have shown that liposomes encapsulating anidulafungin, the latest echinocandin approved by the FDA, are effective against mature *C. albicans* biofilms, reducing metabolic activity to approximately 46% compared to untreated controls over 24 h. Biofilms treated with an equivalent concentration of free anidulafungin did not reduce metabolic activity, further emphasizing the importance of nanoformulations in the treatment of *Candida* biofilms (Vera-González et al., 2020).

In addition to organic nanoparticles, inorganic nanoparticles have also been widely utilized for their antimicrobial properties, most commonly including silver and silica nanoparticles (Cousins et al., 2007; Monteiro et al., 2011; Silva et al., 2013). Silver nanoparticles were recently shown to inhibit biofilm formation of multi-drug resistant *Candida auris* (Lara et al., 2020), an emerging fungal threat with the unique ability to survive on surfaces for several weeks (Welsh et al., 2017). Selenium nanoparticles, which are less toxic to mammalian cells than silver nanoparticles, have only recently been explored for their antimicrobial properties (Huang et al., 2016). Guisbiers et al. (2017) demonstrated that ~100 nm selenium nanoparticles successfully inhibited the formation of *C. albicans* biofilms by attaching to and penetrating through the cell wall (Figure 2: 5B), replacing sulfur with selenium in important biochemical processes. These particles were able to reduce fungal burden in mature biofilms by over 50% at a nanoparticle concentration of as low as 26 ppm (Guisbiers et al., 2017). Inorganic nanoparticles have also been combined with antimicrobial therapeutics, to enhance antifungal properties. de Alteriis et al. (2018) conjugated the mammalian antimicrobial cathelicidin peptide, indolicidin, to the surface of gold nanoparticles (5 nm diameter) in order to protect it from proteolytic degradation and self-aggregation. These particles were able to penetrate and disrupt mature biofilms, eradicating over 50% of the cells for the most *C. albicans* and *C. tropicalis* strains tested after 24 h of treatment when compared to untreated biofilms, with a hypothesized mechanism involving penetration of the fungal cell membrane and inhibition of intracellular targets, arresting cell metabolism (de Alteriis et al., 2018).

## Conclusions and Perspectives

We have discussed several biomaterials strategies from surface functionalization to nanoparticle drug delivery for the prevention and disruption of *Candida* biofilms. Other approaches that can be combined with biomaterials to functionalize surfaces prone to the biofilm formation in the near future include the use of enzymes that target and digest EPS components (Nett, 2014), identification of new drug targets, including inhibition of *Candida* extracellular vesicles (Zarnowski et al., 2018), and incorporation of polymers, such as nylon-3 that have potent and selective activity against *Candida* biofilms (Liu et al., 2014, 2015).

While many advances have been made, development of antifungal biomaterials lags behind the development of antibacterial materials. There is a need for expansion and innovation in antifungal biomaterials, and an emphasis must be placed on advancing technologies beyond preclinical testing. Attention must also be given to polymicrobial biofilms, comprised of multiple fungal and bacterial species, which are currently understudied (Harriott and Noverr, 2009). It is estimated that more than 50% of *C. albicans* infections are polymicrobial in nature (Harriott and Noverr, 2011; Nash et al., 2016). Undoubtedly, it will be critical to use multi-pronged strategies combining effective biomaterials approaches (e.g., surface coatings with nanoparticles) to successfully combat *Candida* and other microbial biofilms.

## Author Contributions

NV-G and AS organized, prepared, and approved the final version of this manuscript. All authors contributed to the article and approved the submitted version.

### Conflict of Interest

The authors declare that the research was conducted in the absence of any commercial or financial relationships that could be construed as a potential conflict of interest.
